# 

**Published:** 1879-09

**Authors:** 


EDITORS:

WILLIAM H. BYFORD, A.M., M.D.,

JAS. NEVINS HYDE, A.M., M.D.   N. S. DAVIS, LL.D., M.D.

DANIEL R. BROWER, M.D.

CHICAGO MEDICAL PRESS ASSOCIATION.

AUG. 2D

1875.

CHICAGO:

*PUBLISHED BY THE CHICAGO MEDICAL PRESS ASSOCIATION.*

188 CLARK STREET.

☞ **Read the Notice of this Journal among Advertisements.**

